# The Mysterious Whistling Breath: Foreign Body Aspiration

**DOI:** 10.7759/cureus.34267

**Published:** 2023-01-27

**Authors:** Sobhashinni Chandran, Boon Chye Gan, Bee-See Goh

**Affiliations:** 1 Otorhinolaryngology-Head and Neck Surgery, Faculty of Medicine, Hospital Canselor Tuanku Muhriz, Kuala Lumpur, MYS; 2 Otorhinolaryngology-Head and Neck Surgery, Island Hospital, Penang, MYS

**Keywords:** emergencies, otorhinolaryngology, bronchoscopy, bronchi, foreign bodies

## Abstract

Foreign body aspiration is commonly seen in the pediatric age group and can be a life-threatening condition. Typical presentations include coughing, wheezing, and choking and can often masquerade as asthma causing misdiagnosis and treatment delay. Most of the time, the actual aspiration event is unnoticed, and patients may remain asymptomatic until they present with recurrent infections with or without positive radiological findings. Aspirated objects tend to migrate distally, and organic objects may induce edema and inflammation. Diagnosis is crucial as near-total or total obstruction of the airway may cause asphyxia and, subsequently, death if no immediate intervention is taken.

## Introduction

Foreign body aspiration can be potentially fatal and requires a multi-disciplinary team approach from emergency, pediatric, anesthesiology, and otorhinolaryngology departments for early diagnosis, optimization, and successful foreign body retrieval. The common clinical presentations include wheezing, dysphonia and stridor [1]. However, these symptoms are also seen in asthma, exacerbation of asthma, croup and pneumonia, which may lead to misinterpretation of the impression and lead to delayed diagnosis [1]. Foreign body aspiration has been the commonest cause of accidental death in children, typically within 1 to 3 years of age [2]. A 60% of complication rate has been reported by Karakoc et al. if the diagnosis was made after 30 days of presentation and no complications if the detection was within 24 hours [2]. Therefore, clinicians must understand that a suspected diagnosis of foreign body aspiration warrants a referral to an otorhinolaryngologist early despite inconcrete symptoms, vague clinical findings, or even a normal chest radiograph [3].

## Case presentation

**Figure 1:**
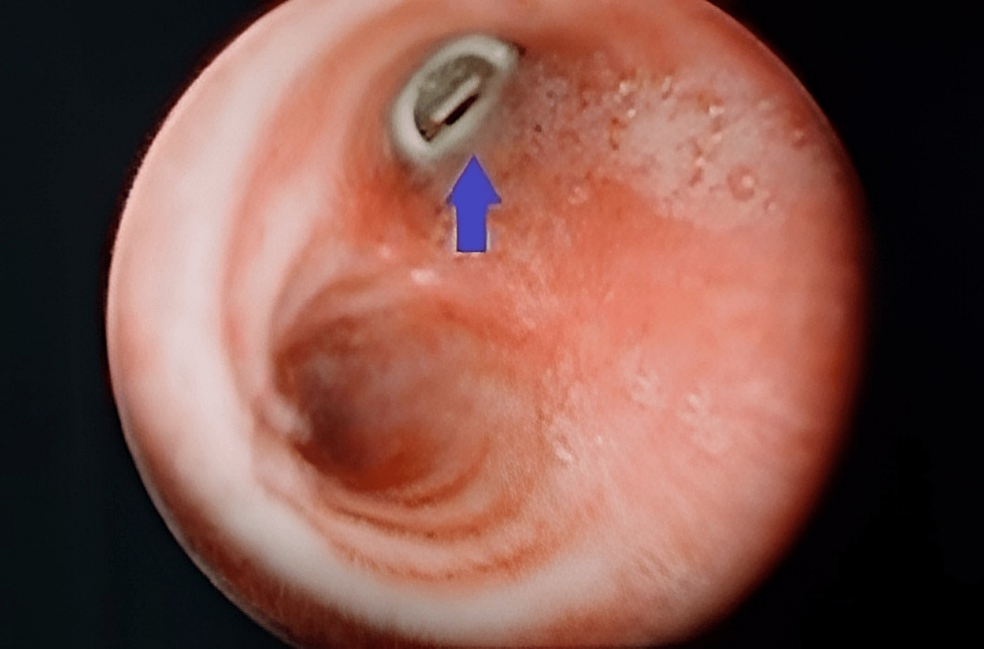
Rigid bronchoscopy showed a foreign body (blue arrow) impacted at the right main bronchus with surrounding edema.

**Figure 2:**
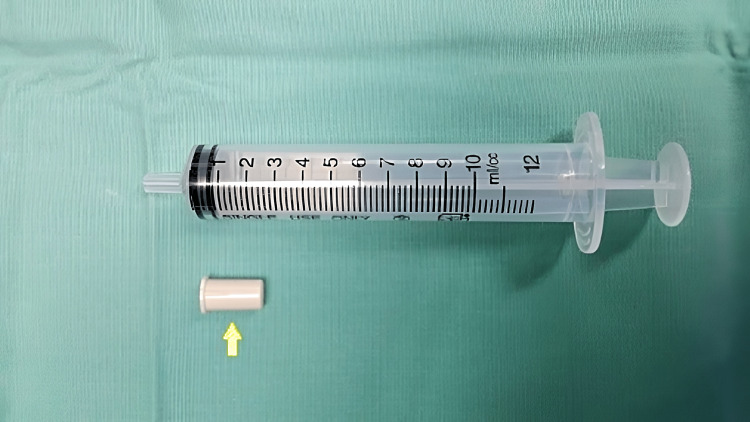
Complete removal of foreign body, a toy blowout whistle (yellow arrow) measuring 1.5cm in length.

**Figure 3:**
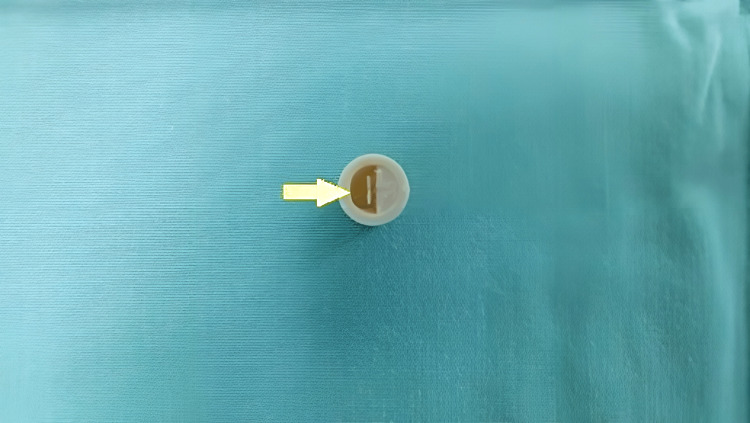
Yellow arrow showing the presence of a semilunar hole acting as a valve in the removed foreign body.

A five-year-old boy presented with a soft abnormal sound mimicking a whistle only upon deep inspiration. The history of initial choking or cough was undetermined, but the mother noted a whistling sound on deep breathing after unsupervised play with his four-year-old cousin. He had no other respiratory symptoms and was comfortable during the examination. Lung auscultation was clear, a chest radiograph was normal, and the flexible scope in the clinic was unremarkable. We proceeded with examination under anesthesia with a direct laryngoscope and rigid bronchoscope. A whitish, cylindrical foreign body with a hole in the center was seen lodged in the right main bronchus (Figure 1). The solid foreign body, which turned out to be a toy blowout whistle (Figures 2, 3), was removed uneventfully using optical forceps. Post-operatively, he was observed for a day and discharged well.

## Discussion

There is often a significant delay in diagnosis of foreign body aspiration due to a lack of good history, and these cases are only diagnosed later when present with complications. A retrospective review done between June 2011 and June 2016 in a Malaysian tertiary academic institution revealed that older children tend to not inform their caretaker of choking episodes which leads to misdiagnosis of asthma [4]. Other misleading initial diagnoses include bronchiolitis, pneumonia, and laryngitis, as reported by Cassol et al. [5]. Complications severity usually depends on the time lag from the onset of aspiration to diagnosis [2]. Often, they may remain asymptomatic until these complications occur. Delayed complications such as lung atelectasis, pneumonia, and bronchiectasis can occur, whereby bronchiectasis is predominantly reported if there is a delay of diagnosis of more than 30 days [2].

According to Sahadan et al., children under the age of three had the highest rate of accidental ingestion and aspiration due to their propensity for placing objects in their mouths, crying, or playing with them while doing so [5]. Due to this habit, another complication that is commonly reported is the impaled foreign body due to the aspiration of the object [6]. The danger imposed is penetrating trauma due to the low resistance of an infant’s tissue to surrounding local barriers [6]. The most prevalent organic foreign bodies were sunflower seeds, peanuts, and other nuts [2]. The largest incidence of inorganic FBA (65.8%) was attributed to the aspiration of needles [2]. Other inorganic objects reported were plastic, wood, or metal [2].

**Figure 4:**
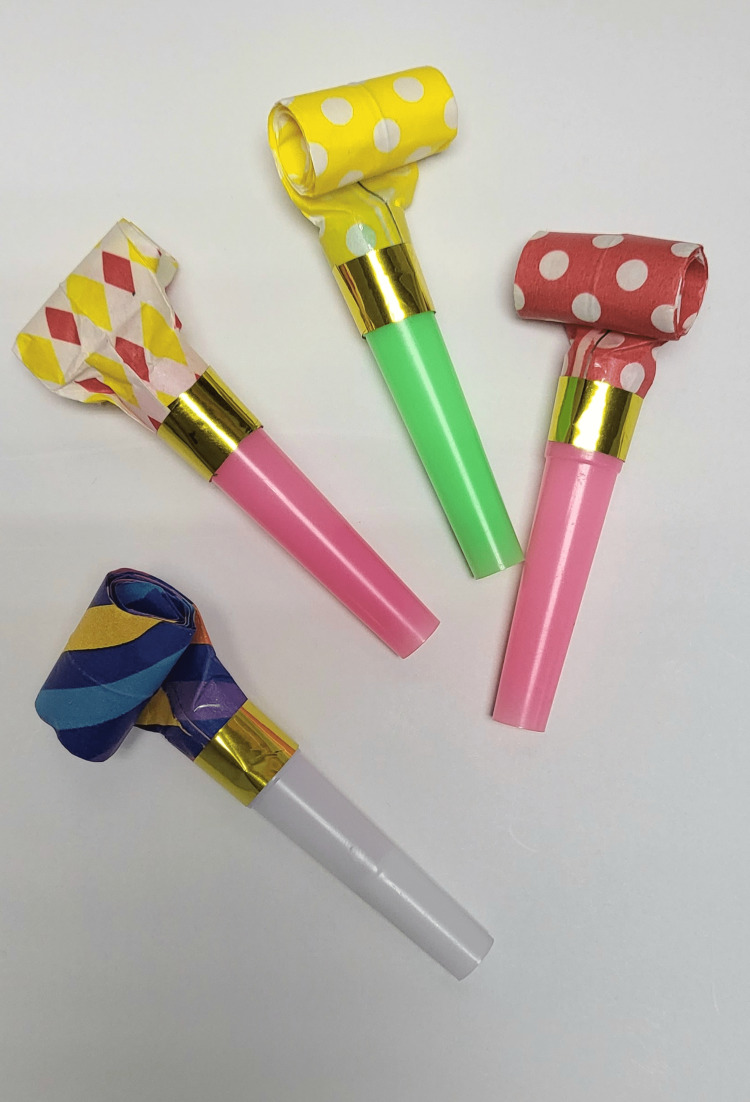
Example of toy blowout whistles.

An initial assessment is usually done with a chest radiograph. The most frequent radiological finding of occlusion of the main bronchus is unilateral atelectasis or emphysema [7]. Despite this, a normal chest X-ray does not rule out foreign body aspiration; hence, a bronchoscopy should always be performed if there is clinical suspicion [3]. Rigid bronchoscopy has been the gold standard method of removal for an aspirated foreign body [5]. Since the right bronchus is anatomically shorter and wider than the left one, it is the location where foreign bodies are most frequently encountered [5]. Early intervention of foreign body removal results in good clinical outcomes with the likelihood of resolution of the complications [3]. While many cases have been reported with positive chest radiograph findings when the foreign body completely occludes the main bronchus, this case was fortunate as the presence of a hole in the toy blowout whistle (Figure 4) and its solid property maintained a patent airway.

## Conclusions

Diagnosis of foreign body aspiration relies importantly on good history taking, thorough physical examination, and high index of suspicion even when there is no eyewitness and positive radiological findings. A high degree of suspicion is mandated, particularly when a choking event is not clear in history. Clinicians should maintain a low threshold for bronchoscopy in the pediatric age group despite normal radiological findings.
